# Leaf-damaging behavior by queens is widespread among bumblebee species

**DOI:** 10.1038/s42003-025-07670-3

**Published:** 2025-03-14

**Authors:** Priska Flury, Sofie Stade, Consuelo M. De Moraes, Mark C. Mescher

**Affiliations:** Department of Environmental Systems Sciences, ETH Zürich, Zürich, Switzerland

**Keywords:** Behavioural ecology, Plant ecology, Animal behaviour, Entomology

## Abstract

Phenological mismatches and resource limitations resulting from ongoing environmental change can have severe impacts on pollinator fitness. Recent findings show that bumblebee workers respond to pollen scarcity by damaging plant leaves in ways that can accelerate flowering, suggesting a mechanism by which direct information transfer from bees to plants might influence the timing of flower production. However, the ecological and adaptive significance of this interaction remains uncertain. Here we report that mated and unmated queens of *Bombus terrestris* also damage leaves, with similar effects on flowering. Furthermore, we document leaf damage by wild-caught queens from 12 species, spanning seven subgenera, indicating damaging behavior is widespread among *Bombus* species. Leaf damage by bumblebee queens may have particular relevance in the context of colony founding and early development, where the timely availability of local floral resources can be critical for colony success and fitness.

**Figure:**
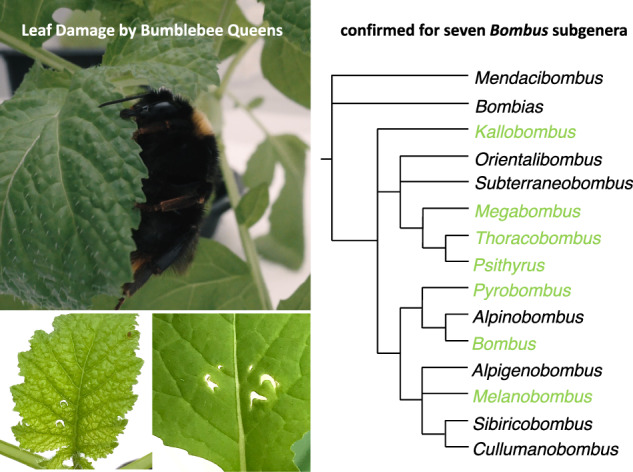

## Abstract (web summary)

Experiments with captive and wild queens show that leaf-damaging behavior is widespread among bumblebee species and can accelerate plant flowering.

## Introduction

Temporal synchrony between plants and pollinators may be disrupted by ongoing environmental change, including climate warming^1,2^. The resulting phenological mismatches can have adverse impacts on pollinator fitness, particularly for species with annual reproductive cycles, including bumblebees, which rely heavily on the timely availability of floral resources during critical developmental stages^3–7^. Recently, we reported that bumblebee (*Bombus terrestris*) workers respond to pollen scarcity by damaging plant leaves and that the damage inflicted by bumblebees can accelerate plant flowering^8^. This discovery reveals a mechanism by which the timing of flower production can be influenced by direct interaction between plants and pollinators, and we hypothesized that damage-induced acceleration of flowering might benefit bumblebees at early stages of colony development, as well as during temporal gaps in flower availability later on^8^. Yet, the broader ecological and adaptive significance of bumblebee leaf-damaging behavior remains uncertain, and key features of the behavior itself remain to be characterized, including patterns of occurrence at the level of individual bees, colonies, and species^8^.

Bumblebees are important pollinators in both natural and agricultural ecosystems and face growing challenges from anthropogenic stressors, including climate change^6,9,10^. Effects of bumblebee leaf damage on flowering could be particularly important in the context of colony founding, as the availability of such resources in the immediate proximity of founding nests strongly influences colony survival and performance^7,11,12^; moreover, fine-scale variation in the timing of resource availability during this critical period (on the order of a few days) can have large impacts on colony development^13^. Newly mated bumblebee queens overwinter in isolation and typically found new colonies alone in early spring^14^. During the initial, workerless phase of colony development, queens are solely responsible for provisioning and rearing brood and are known to perform diverse tasks within and outside the nest, including foraging for floral resources^14–16^. While leaf-damaging behavior by queens has not previously been reported, it could plausibly have fitness benefits in this context. Because leaf-damaging behavior is linked to pollen availability^8^, damaged-induced acceleration of flowering may also enhance colony performance by helping to mitigate temporal gaps in flower availability at later stages of colony development^17,18^. Damaging behavior by bumblebee queens could again be relevant here, as young queens have been reported to engage in other foraging activities outside the natal nest prior to mating^19,20^. Given the plausible fitness benefits of leaf damage by queens, a key goal of the current study was to determine whether young bumblebee queens damage plant leaves and thereby influence plant flowering time.

As discussed below, initial experiments with mated and unmated *B. terrestris* queens reared in the laboratory confirmed that queens do indeed damage plant leaves and that the damage inflicted can accelerate plant flowering, similar to effects previously demonstrated for *B. terrestris* workers^8^. This finding presented a unique opportunity to conduct behavioral experiments with wild-caught queens in order explore the taxonomic distribution of damaging behavior across *Bombus* species. These experiments confirmed that queen-damaging behavior is present in at least 12 species, spanning seven *Bombus* subgenera, including species exhibiting considerable variation in natural history and ecology.

## Results and discussion

### *Bombus terrestris* queen inflicted leaf-damage accelerates flower production

**Fig. 1:**
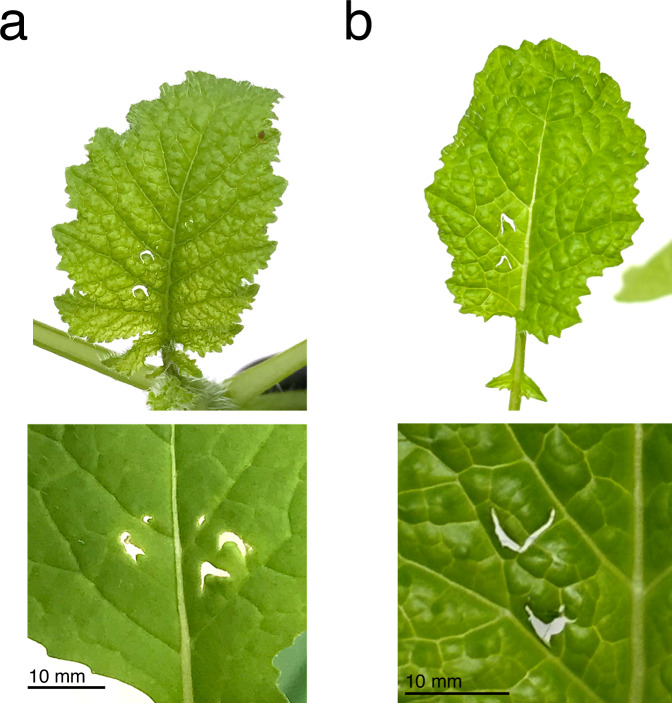
Typical leaf damage by unmated queens of *Bombus terrestris.* **a** Typical example of leaf damage by unmated *B. terrestris* queens on *B. nigra* plants (scale bar = 10 mm). **b** Mechanical reproduction of queen damage using needles on *B. nigra* plants (scale bar = 10 mm).

**Fig. 2:**
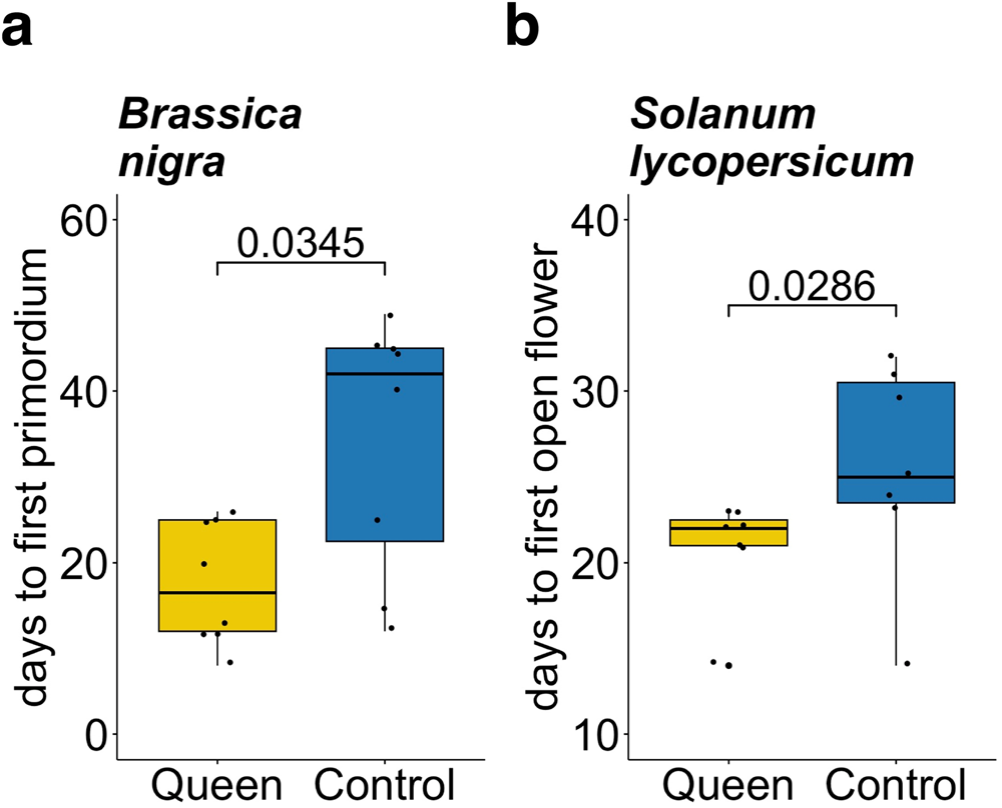
Effects of queen damage on time to flower of *Brassica nigra* and *Solanum lycopersicum.* Reproductive development was monitored for **a** *B. nigra* and **b** *S. lycopersicum* plants assigned to two treatments: (i) unmated queen damage and (ii) mechanical damage (control). Following damage treatment, plants were monitored daily for macroscopic signs of flowering (indicated on the vertical axes, days post treatment). Damage by unmated *B. terrestris* queens accelerated the average vegetative-to-reproductive switch of *B. nigra* plants by 17 days relative to mechanically damaged controls (*n* = 8 biologically independent samples) and the flowering time of *S. lycopersicum* plants by 5 days (*n* = 7 biologically independent samples). Data is shown as boxplots, where the line represents the median and the box the interquartile range (IQR). A pairwise comparison (Exact Wilcoxon-Mann-Whitney Test) was used to determine statistical significance. The *p*-value is indicated above brackets.

Initial observations from growth-chamber experiments revealed that recently eclosed, unmated *B. terrestris* queens exhibit damaging behavior under conditions of pollen-deprivation similar to that of workers; moreover, damage by queens typically created the same characteristic pattern of crescent-shaped holes previously reported for *B. terrestris* workers (Fig. 1a, b)^8^. This behavior also persisted in queens subjected mating and simulated overwintering via induced hibernation. Subsequent experiments revealed that queen damage by unmated queens also has significant effects on plant reproductive development relative to similar patterns of mechanically inflicted damage (Fig. 2a, b).

### Leaf-damaging behavior is widespread among *Bombus* subgenera

**Fig. 3:**
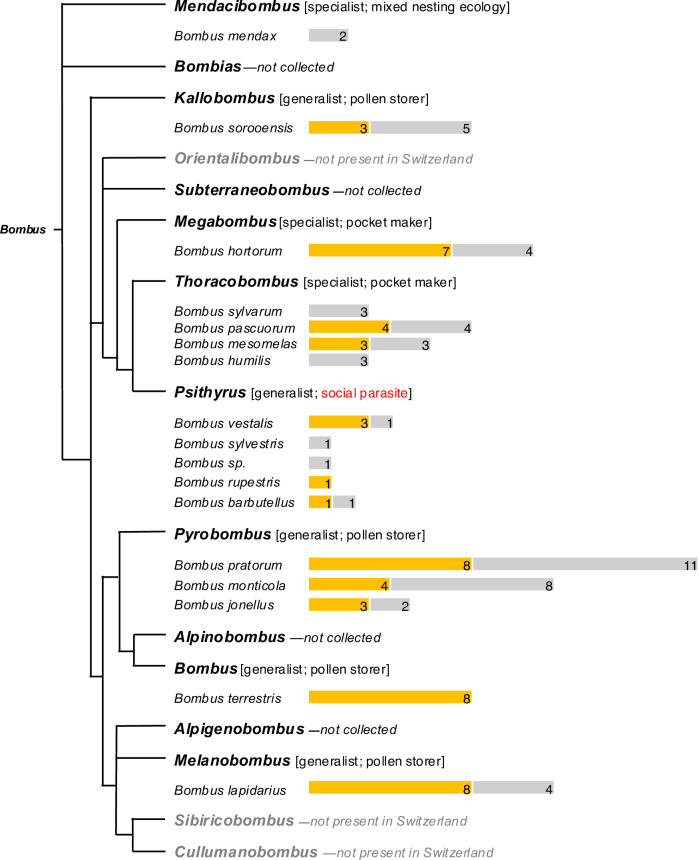
Distribution of queen damaging behavior across *Bombus* subgenera and species. Simplified phylogenetic tree of *Bombus* subgenera, adapted from Williams et al. ^21^. Subgenera are indicated in bold text, with collected species listed below (gray text indicates subgenera not found in Switzerland). For each species, yellow bars represent numbers of damaging individuals and gray bars represent individuals that were not observed to damage. Information about host-plant specialization/ dietary breadth (as indicated by tongue length^31^) and nesting ecology for each subgenus are presented in brackets (social parasitism is highlighted in red). *Bombus sp*. = identification only to subgenus level. *B. terrestris* queens came from a commercial colony and were artificially hibernated.

The observation that *B. terrestris* queens damage leaves suggested that similar behavioral assays with queens of other species could provide an effective method for exploring the taxonomic distribution of damaging behavior, which is otherwise challenging because of the difficulty in rearing colonies of most wild species. To determine whether queens of other Swiss bumblebee species also damage leaves, we collected queens in flight from several field sites in the Swiss Alps, beginning in early spring of 2023. Based on timing and ecological context, we can be confident these were mated queens that had recently emerged from hibernation and presumably were in the process of founding new colonies (see methods). These collections yielded 105 queens from 17 bumblebee species across 8 *Bombus* subgenera (Fig. 3). Worldwide, the genus *Bombus* compromises over 250 extant species across 15 subgenera^21^, of which 41 species and 12 subgenera occur in Switzerland^22^. Our collections thus represent a broad sample of Swiss taxa and include species from a majority of *Bombus* subgenera. Subsequent assays confirmed leaf-damaging behavior by queens from 12 species belonging to 7 subgenera (Fig. 3). Moreover, while we did not observe leaf-damaging behavior in every species examined, species for which the behavior was not recorded invariably included observations from only a small number of individuals (1–3). Thus, while our results conclusively show that leaf-damaging behavior is widespread among *Bombus species* occurring in Switzerland, they are also consistent with the possibility that the behavior is ubiquitous within the genus. In addition to our behavioral assays, we also observed characteristic patterns of leaf damage, consistent with that inflicted by  bumblebees on wild growing plants at our field sites. The absence of bumblebee workers—and the limited presence of other insects—in our field sites at the time of these observations further increases our confidence that this damage was caused by bumblebee queens and suggests that queens do indeed damage plant leaves in the context of colony founding.

Of the 12 species for which leaf-damaging behavior by queens was documented, we have evidence to date confirming damage by workers for four species: In addition to *B. terrestris*, which we routinely use for damaging experiments in the laboratory and field, we previously reported observations of leaf damage by workers of *B. lucorum* and *B. lapidarius*^8^. In the current study we also observed damaging behavior by a microcolony comprising wild-caught workers of *B. pratorum* (Fig.S1). We were unable to successfully establish microcolonies with wild caught workers of other species. However, available data are again consistent with the hypothesis that damaging behavior is widespread among *Bombus* species.

**Fig. 4:**
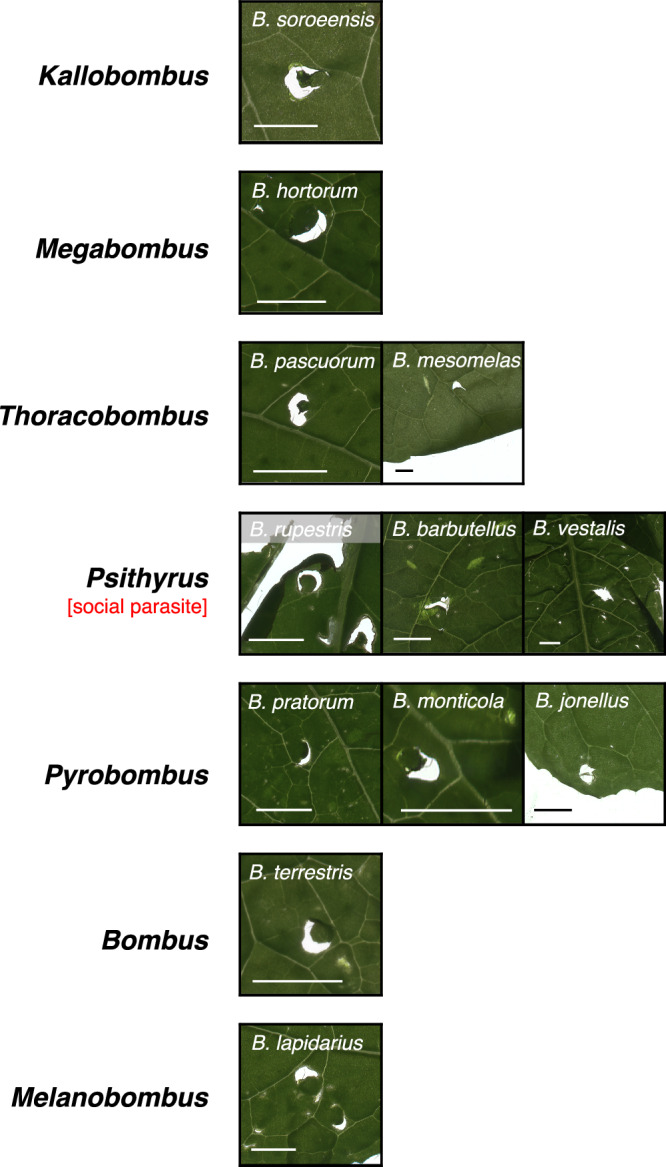
Variation of leaf-damage shape in different Bombus species. Representative scans of leaves damaged by spring queens (mated and hibernated) of each species (scale-bar = 3 mm). Plants used for this experiment were *Brassica nigra* and *Solanum lycopersicum*.

The taxonomic breadth of our queen collections allowed us also to examine variation in damaging behavior among species exhibiting differences in ecology and natural history. As we hypothesized that damaging behavior by founding queens has fitness benefits deriving from effects on floral resource availability, we predicted that damaging behavior might be absent or divergent in socially parasitic (cuckoo) species in the subgenus *Psithyrus*, where queens invade already established colonies of other species and do not produce their own workers, although the host colony is still depending on pollen availability^14^. Our behavioral assays confirmed damaging behavior by queens from three of the five *Psithyrus* species captured in our survey; however, queens from two of these species consistently produced an atypical damage pattern and were the only species in our study not observed to create the characteristic crescent-shaped holes previously reported for *B. terrestris* workers^8^ (Fig. 4). Unfortunately, we were unable to determine whether this divergent damage pattern has effects on flower production, as the limited numbers of queens collected from the field were not sufficient for flowering-time assays. We further hypothesized that leaf-damaging behavior and its effects on flowering-time might be more important for alpine species occurring at higher elevation, which can be more vulnerable to mismatched timing of early season flowering time and worker activity^6,16^; however, we observed damaging behavior in both high- and low-elevation species. Moreover, we observed damaging behavior in species exhibiting different nesting ecology (pocket makers, pollen storers and social parasites) and different levels of plant specialization (long vs. short tongued species) (Fig. 3).

### Implications for the ecological and adaptive significance of leaf-damaging behavior

In summary, our results show that (i) young, mated and unmated, bumblebee queens damage plant leaves, (ii) queen damage, like worker damage, can accelerate flowering; and (iii) leaf-damaging behavior is present in a wide range of species spanning most *Bombus* subgenera and considerable variation in ecology, natural history, and social organization. Taken together, these findings indicate that leaf damaging is a typical component of the behavioral repertoire of many, and perhaps all, bumblebee species. In future studies we hope to examine damaging behavior in *Bombus* species from other global regions, including new-world species such as *Bombus impatiens*, as well as to other closely related taxa, including the stingless bees (Meliponini).

The widespread distribution of leaf-damaging behavior strongly suggests that it has adaptive significance for bumblebees, plausibly linked to its observed effects on flowering time. Moreover, the observation that mated bumblebee queens damage plant leaves in the context of colony founding is consistent with our hypothesis that damage-induced effects on flower production play a role in enhancing local resource availability during early stages of colony development^8^, when fine-scale variation in the spatio-temporal availability of resources are likely to have critical impacts on colony survival and success^7,11,12^. As noted above, the maintenance of temporal synchrony with flowering plants is a perennial challenge for pollinating species. In contrast to known herbivory-induced plant responses, which often negatively affects plant flowering^23–25^, bumblebee damage-induced positive effects on flowering time represent a potential mechanism by which direct information transfer between pollinators and plants may fine-tune the timing of flower production. The existence of such a mechanism may have particular ecological relevance in the context of ongoing climate change or other rapid and environmental shifts in environmental conditions that might disrupt other mechanisms for the maintenance of synchrony.

## Methods

### Plants and insects

#### Commercial *Bombus terrestris* queens

Unmated *Bombus terrestris* queens were obtained from commercially available queenright colonies provided by the Biobest group NV (Westerlo, Belgium). Nests were provided with BIOGLUC® sugar solution (2.1 kg) and fed with lyophilized pollen (Bio-Blütenpollen, naturwaren-niederrhein GmbH, Germany) provided either as pure ground powder or mixed with nectar. Colonies were maintained in climate chambers in complete darkness (Kälte 3000: day/night cycle: 16:8, 24 h darkness; RH70%/80%; 27/26 °C). Founding queens were removed after we received the hives, in order to accelerate the production of new queens from developing brood as described by Lopez-Vaamonde et al.,^26^ (colonies arrived 27.04.2022; new queens eclosed 18.05.2022). Queens were readily distinguishable from workers based on size. Newly eclosed gynes were isolated and kept in small cages (W60 × D40 × H40 cm) in a climate chamber (Kälte 3000: light/dark cycle: 12:12, 150 μE; RH60%/70%; 22/18 °C;). Queens were deprived of pollen 3 days prior to experiments. A separate batch of unmated queens from commercially obtained colonies were mated with males (from a different commercially obtained colony) and used to confirm the persistence of damaging behavior following experimentally induced hibernation, using methods described by Röseler^27^.

#### Wild bumblebee queens

Wild bumblebee queens were caught using insect nets in Spring 2023 (end of February 2023 until end of July 2023). We collected queens from different regions of Switzerland (Fig.S2) and from different elevations: Haldenstein (Calanda 1400 m a.s.l., 2000 m a.s.l.), greater Zurich area (City Center 400 m a.s.l., Uetliberg 870 m a.s.l.), Baden 500 m a.s.l., Landquart 550 m a.s.l., Niederwil 405 m a.s.l. Delayed phenology at higher elevation shifted the timing of queen emergence to mid-July (Snow melt dates for 2000 m asl: mid-May). To avoid collecting queens that had already initiated a colony, we only kept queens without pollen bags and stopped collecting queens as soon as we observed flying workers at each collection site. Queens were anesthetized with CO_2_ for 1 min and identified under a microscope using the *Fauna Helvetica Apidae 1* identification guide for Swiss bumblebees^22^. Individual queens were kept in small enclosures (W30 × D30 × H30 cm) in a climate chamber (Kälte 3000: light/dark cycle: 12:12, 150 μE; RH60%/70%; 22/18 °C) for at least 2 weeks and given access to nectar (Biogluc sugar solution, Biobest group) *ad libitum*. Pollen (Bio-Blütenpollen, naturwaren-niederrhein GmbH, Germany) was provided once a week, but during exposure to plants no pollen was available to ensure conditions of pollen-deprivation.

#### Plants

*Brassica nigra* plants were grown from seed provided by the Center of Genetic Resources in Wageningen, the Netherlands (accession number: CGN06619) and further propagated in the lab. Seeds were sown in plastic trays with “substrate 2 + greenfibre (120)” as soil (Klasmann-Deilmann GmbH, Geeste, Germany) and irrigated with water mixed with ~0.25% (v/v) Solbac (Andermatt Biocontrol Suisse AG, Grossdietwil, Switzerland). Trays were stored in darkness for 3 days at 4 °C to synchronize germination and subsequently kept in a climate chamber (Kälte 3000, light/dark cycle: 12:12, 150 μE; RH60%/70%; 22/18 °C). One week after germination, seedlings were transplanted into single pots (Desch PlantPak, 9 × 9 × 10 cm) and treated with ~0.25% (v/v) Solbac (Andermatt Biocontrol Suisse AG, Grossdietwil, Switzerland). *Solanum lycopersicum* var. Sibirian Early 34500 (tomato) plants were grown from seed obtained from Zollinger bio (Port-Valais, Switzerland). Seeds were sown in plastic trays filled with “substrate 2 + greenfibre (120)” (Klasmann-Deilmann GmbH, Geeste, Germany) and watered with water mixed with ~0.25% (v/v) Solbac (Andermatt Biocontrol Suisse AG, Grossdietwil, Switzerland). Trays were kept in a climate chamber (Kälte 3000: light/dark cycle: 16:8, 150 μE; RH60%/70%; 22/18 °C). One week after germination seedlings were transplanted into single pots (Desch PlantPak, 9 × 9 × 10 cm) and treated with ~0.25% (v/v) Solbac (Andermatt Biocontrol Suisse AG, Grossdietwil, Switzerland).

### Experimental methods

#### Flowering-time assays with *B. terrestris* queens

In both flowering-time experiments, plants were randomly assigned to two treatment groups: queen-damage and mechanical damage (control) (*B. nigra*, *n* = 8 per treatment; *S. lycopersicum*, *n* = 7 per treatment). Each queen-damaged plant was paired with a mechanically damaged control plant, on which we tried to replicate the damage pattern as closely as possible using needles (Fig. 1a, b). Findings from our previous study^8^ indicate that mechanical damage can cause slight acceleration of flowering in *S. lycopersicum*, but that this is effect is consistently weaker than that observed for damage by *B. terrestris* workers. All plants were of uniform age at the start of the experiment (*B. nigra*, 24 days post germination (dpg); *S. lycopersicum*, 25 dpg) and had the same number of leaves (*B. nigra*, 8 true leaves; *S. lycopersicum*, 9 true leaves). Plants in the queen-damage treatment were placed into cages with individual, pollen-deprived queens (*B. terrestris* queens, *n* = 4, 2 or 3 plants per queen) in a climate chamber (Kälte 3000: light/dark cycle: 12:12, 150 μE; RH60%/70%; 22/18 °C). It took between several minutes and 24 h for queens to begin damaging individual plants, and plants were removed once queens were observed having damaged a plant. On average, *Brassica* plants received 9 ± 7 holes, *Solanum* plants received 2 ± 1. After treatment, plants were placed at random positions in their respective climate chamber (*B. nigra*, LD 12:12; *S. lycopersicum*, LD 16:8). Trays were moved to new random positions every 3 days. Plants were monitored daily for the development of macroscopic signs of flowering (*B. nigra*, first flowering primordium; *S. lycopersicum*, first open flower), and flowering time was assessed as “time (days) elapsed since damage treatment”.

#### Leaf-damaging assays with wild bumblebee queens

Individual flowerless plants of *Brassica nigra* (20–30 days post germination) or *Solanum lycopersicum* (30–40 days post germination) were placed in enclosures with individual wild bumblebee queens in a climate chamber (Kälte 3000, light/dark cycle: 16:8, 150 μE; RH60%/70%; 22/18 °C). The plant species a queen received was based on availability. Plants were inspected daily for new leaf-damage. Damage was documented by cutting damaged leaves and scanning them using the Epson Perfection V850 Pro Scanner. The following settings were applied in the Epson Scan Software: document type: film; film type: color positive film; image type: 24-bit color; resolution: 1200 dpi; adjustments: none; file type: TIFF. Leaves were scanned in batches every 2–3 days.

#### Leaf-damaging assays with wild bumblebee workers

In May 2023, we started collecting wild foraging workers from different species to form microcolonies, which could be used to test leaf-damaging behavior of the worker caste. 10–20 worker per species, originating from different colonies, were collected in the center of Zurich, kept together in a plastic box with sufficient ventilation in a climate chamber (Kälte 3000: day/night cycle: 16:8, 24 h darkness; RH60%/70%; 22/18 °C), and given around 10 days to establish a new hierarchy. Only *B. pratorum* workers formed a microcolony that could be used for damaging experiments. Microcolonies are usually formed by separating workers from their parental colony. Our method provides a way to circumvent the time-consuming process of rearing a colony first from wild queens. The colony was pollen-deprived for 3 days before being moved into a cage (W60 × D60 × H60 cm) in a climate chamber (Kälte 3000: light/dark cycle: 16:8, 150 μE; RH60%/70%; 22/18 °C) and presented with flowerless *S. lycopersicum* plants. Damaged plants were removed from the cage and the leaves were scanned as described above. A selection of leaf scans is found in the supplementary material.

### Statistical analyses

All statistical analyses were carried out using R version 4.3.1 (R Core Team 2023)^28^. For flowering-time experiments, displayed in Fig. 2, we used an exact, two-sided Wilcoxon-Mann–Whitney Test to test for the equality of means between two independent groups. We used the function “wilcox_test ()” from the R package *coin*. Boxplots were plotted using the “ggboxplot ()” function from the *ggplot2* package and *p*-values were added to the plot using the function “stat_pvalue_manual ()” function form the package *rstatix* and the *p*-values were calculated using the function “dunn_test ()” also from the package *rstatix*. A summary of the statistics can be found in the supplementary table 1 (ST1).

### Ethical note

In Switzerland, there is no legislation regulating research with bumblebees; however, our experimental design and procedures were guided by the 3 R principles^29^. Bees received daily care by trained staff and were provisioned with adequate food. Behavioral tests were non-invasive, and we tried to minimize stress wherever possible.

### Reporting summary

Further information on research design is available in the Nature Portfolio Reporting Summary linked to this article.

## Supplementary information

Supplemental Information

Reporting summary

## Data Availability

All raw data that support the findings of this study are available in the online repository on Figshare (10.6084/m9.figshare.26190911.v2)^30^.

## Code availability

The R-code and the respective.txt data files, but also the raw-data excel sheets can be found in the online repository on Figshare (10.6084/m9.figshare.26190911.v2)^30^.
