# Supplementary material for: Leaf-damaging behavior by queens is widespread among bumblebee species
Source: Commun Biol. 2025 Mar 14;8:435. doi: 10.1038/s42003-025-07670-3 (PMC11906820; doi:10.1038/s42003-025-07670-3)
Supplement: Supplementary file 1 — Supplemental Information [file 42003_2025_7670_MOESM1_ESM.pdf]

Supplementary Material

Leaf-damaging behavior by queens is widespread among bumblebee species

Priska Flury[1], Sofie Stade[1], Consuelo M. De Moraes[1] †, Mark C. Mescher[1] †

[1]Department of Environmental Systems Sciences, ETH Zürich, Zürich, Switzerland.

† Corresponding authors. Email: mescher@usys.ethz.ch; consuelo.demoraes@usys.ethz.ch

Supplementary figure 1: Leaf damage of wild *Bombus pratorum* queens and workers.
Supplementary figure 2: Pictures of field site in Swiss Alps.
Supplementary table 1: Summary statistics.

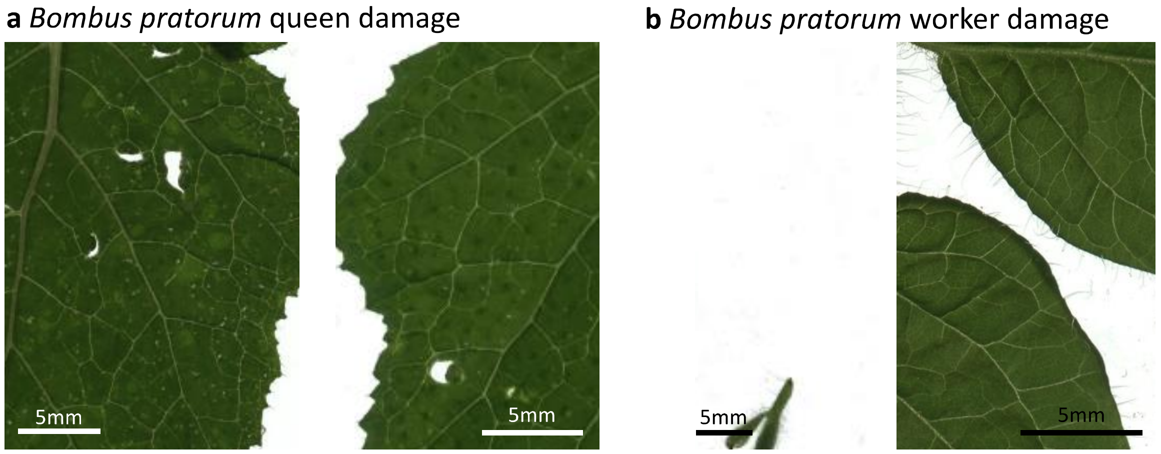

Fig.S1.: Leaf damage of wild *Bombus pratorum* queens and workers.
a, Typical leaf damage of a *B.pratorum* queen on *Brassica nigra*. **b**, Typical leaf damage of a *B.pratorum* worker on *Solanum lycopersicum*. Scale bar=5mm.

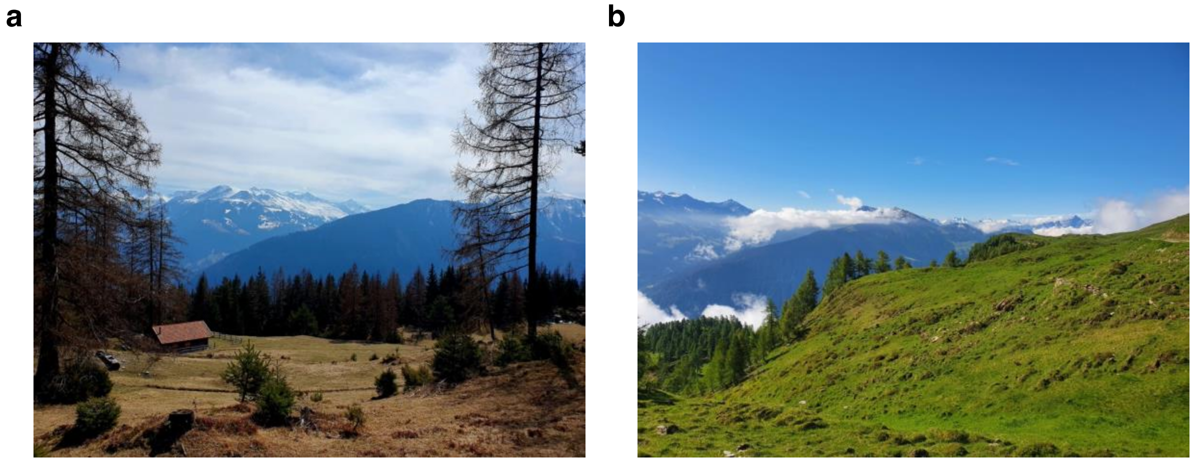

Fig.S2.: Wild queen collection sites.
In Spring 2023 (end of February 2023 until end of July 2023) we collected wild bumblebee queens at different locations in Switzerland. Many of our collections happened at a field site in the Swiss mountains mainly because the delayed phenology at higher elevation shifted the timing of queen emergence to mid-April/mid-May at 1400m and mid-July for 2000m. **a**, Sampling site in Haldenstein, Switzerland (Calanda mountain, 1400m) **b**, Sampling site in Haldenstein, Switzerland (Calanda mountain, 2000m).

**plant species**	***Brassica nigra***	***Solanum lycopersicum***
**biological replicates per treatment**	8	7
**mean *duf* queen**	17.6±7.2	20.86±3.13
**mean *duf* mechanical**	34.38 ±14.8	25.57±6.24
**Exact Wilcoxon-Mann-Whitney Test wilcox_test()**		
**Z-value**	2.1145	2.1891
**p-value**	0.03419	0.02972
**Package *rstatix* dunn_test ()**		
**statistic**	-2.11	-2.19
**p.adjusted**	0.0345	0.0286

Supplementary Table 1: Summary statistics.

For both plant species, the mean days until flowering (duf) was calculated for both treatments (queen= plants damaged by unmated queens, mechanical= mechanical replication of the damage). Statistical significance was determined by exact Wilcoxon-Mann-Whitney test using the function wilcox_test from the R package *coin*. The Z- and p-value are shown in the table. For visualization, we used the function stat_pvalue_manual() from the package *rstatix* and the p-values for this were calculated using the function dunn_test() also from the package *rstatix*. Also here the relevant statistics and adjusted p-vlaues are shown in the table. The R-code can be found in the online repository on figshare (see code availability).
