# Supplementary material for: Leaf-damaging behavior by queens is widespread among bumblebee species
Source: Commun Biol. 2025 Mar 14;8:435. doi: 10.1038/s42003-025-07670-3 (PMC11906820; doi:10.1038/s42003-025-07670-3)
Supplement: Supplementary file 2 — Reporting summary [file 42003_2025_7670_MOESM2_ESM.pdf]

nature portfolio

Corresponding author(s): Mark Mescher

Last updated by author(s): Jan 24, 2025

Reporting Summary

Nature Portfolio wishes to improve the reproducibility of the work that we publish. This form provides structure for consistency and transparency in reporting. For further information on Nature Portfolio policies, see our Editorial Policies and the Editorial Policy Checklist.

Statistics

For all statistical analyses, confirm that the following items are present in the figure legend, table legend, main text, or Methods section.

n/a	Confirmed	
☐	☒	The exact sample size (n) for each experimental group/condition, given as a discrete number and unit of measurement
☐	☒	A statement on whether measurements were taken from distinct samples or whether the same sample was measured repeatedly
☐	☒	The statistical test(s) used AND whether they are one- or two-sided<br>*Only common tests should be described solely by name; describe more complex techniques in the Methods section.*
☒	☐	A description of all covariates tested
☒	☐	A description of any assumptions or corrections, such as tests of normality and adjustment for multiple comparisons
☐	☒	A full description of the statistical parameters including central tendency (e.g. means) or other basic estimates (e.g. regression coefficient) AND variation (e.g. standard deviation) or associated estimates of uncertainty (e.g. confidence intervals)
☐	☒	For null hypothesis testing, the test statistic (e.g. F, t, r) with confidence intervals, effect sizes, degrees of freedom and P value noted<br>*Give P values as exact values whenever suitable.*
☒	☐	For Bayesian analysis, information on the choice of priors and Markov chain Monte Carlo settings
☒	☐	For hierarchical and complex designs, identification of the appropriate level for tests and full reporting of outcomes
☒	☐	Estimates of effect sizes (e.g. Cohen's d, Pearson's r), indicating how they were calculated

Our web collection on statistics for biologists contains articles on many of the points above.

Software and code

Policy information about availability of computer code

Data collection	No software was used to collect data.
Data analysis	Data was analysed using R-software version 4.3.1 (R Core Team 2023). The R-code and the respective .txt data files, but also the raw-data excel sheets can be found in the online repository on figshare (https://doi.org/10.6084/m9.figshare.26190911.v2)

For manuscripts utilizing custom algorithms or software that are central to the research but not yet described in published literature, software must be made available to editors and reviewers. We strongly encourage code deposition in a community repository (e.g. GitHub). See the Nature Portfolio guidelines for submitting code & software for further information.

Data

Policy information about availability of data

All manuscripts must include a data availability statement. This statement should provide the following information, where applicable:

- Accession codes, unique identifiers, or web links for publicly available datasets
- A description of any restrictions on data availability
- For clinical datasets or third party data, please ensure that the statement adheres to our policy

All data that support the findings of this study are available on figshare (https://doi.org/10.6084/m9.figshare.26190911.v2).

March 2021

Human research participants

Policy information about studies involving human research participants and Sex and Gender in Research.

Reporting on sex and gender	*Use the terms sex (biological attribute) and gender (shaped by social and cultural circumstances) carefully in order to avoid confusing both terms. Indicate if findings apply to only one sex or gender; describe whether sex and gender were considered in study design whether sex and/or gender was determined based on self-reporting or assigned and methods used. Provide in the source data disaggregated sex and gender data where this information has been collected, and consent has been obtained for sharing of individual-level data; provide overall numbers in this Reporting Summary. Please state if this information has not been collected. Report sex- and gender-based analyses where performed, justify reasons for lack of sex- and gender-based analysis.*
Population characteristics	*Describe the covariate-relevant population characteristics of the human research participants (e.g. age, genotypic information, past and current diagnosis and treatment categories). If you filled out the behavioural & social sciences study design questions and have nothing to add here, write "See above."*
Recruitment	*Describe how participants were recruited. Outline any potential self-selection bias or other biases that may be present and how these are likely to impact results.*
Ethics oversight	*Identify the organization(s) that approved the study protocol.*

Note that full information on the approval of the study protocol must also be provided in the manuscript.

Field-specific reporting

Please select the one below that is the best fit for your research. If you are not sure, read the appropriate sections before making your selection.

☐ Life sciences ☐ Behavioural & social sciences ☒ Ecological, evolutionary & environmental sciences

For a reference copy of the document with all sections, see nature.com/documents/nr-reporting-summary-flat.pdf

Ecological, evolutionary & environmental sciences study design

All studies must disclose on these points even when the disclosure is negative.

Study description	Here we report the leaf damaging behavior of mated and unmated queens of Bombus terrestris. Furthermore, we show that damaging behavior is widespread among Bombus species, documenting damage by wild-caught queens from 12 species, spanning seven subgenera.
Research sample	We used female individuals of several species of bumblebees (genus Bombus). We list all used species here:<br>Bombus terrestris<br>Bombus mendax<br>Bombus soroeensis<br>Bombus hortorum<br>Bombus sylcarum<br>Bombus pascuorum<br>Bombus mesomelas<br>Bombus humilis<br>bombus vestalis<br>Bombus sylvestris<br>Bombus spp.<br>Bombus rupestris<br>Bombus barbutellus<br>Bombus pratorum<br>Bombus monticola<br>Bombus jonellus<br>bombus terrestris<br>Bombus lapidarius<br>Bombus wurflenii<br>Bombus ruderatus
Sampling strategy	We collected wild individuals in early spring at different field sites. The sample size for each species was dependent on availability of caught individuals. For B. terrestris, commercial colonies were used to rear new queens. Also here, the sample size of four queens is limited by availability. On the plant side, several of our experiments showed that a sample size of 8 per treatment is sufficient to see the effect of leaf-damaging on flowering time.
Data collection	For flowering-time studies:<br>Two flowering-time experiments were conducted using two different plant species: Brassica nigra (n= 8 plants per treatment) and Solanum lycopersicum (n=7 plants per treatment). Individual plants were assigned randomly to two treatment groups: queen-

damage and mechanical damage (control. We paired experimental plants, so that each queen-damaged plant had a control plant, where we tried to mimick the exact damage pattern as closely as possible by using needles. The plants were of uniform age at the beginning of the experiment ((B. nigra, 24 days post germination (dpg); S. lycopersicum, 25dpg) and had the same number of leaves (B. nigra, 8 true leaves; S. lycopersicum, 9 true leaves). Experimnetla plants were exposed to pollen-deprived queens (B.terrestris, n=4, 2-3 plants per queen). Queens and plants were placed together in cages in a climate chamber (Kälte 3000: light/dark cycle: 12:12, 150 µE; RH60%/70%; 22/18°C). It took between several minutes and 24hours for queens to begin damaging individual plants, and plants were removed once queens were observed having damaged a plant. On average, Brassica plants received 9±7 holes, Solanum plants received 2±1. In the following weeks,the plants were monitored daily for the development of macroscopic signs of flowering (B. nigra, first flowering primordium; S. lycopersicum, first open flower), and flowering time was assessed as "time (days) elapsed since damage treatment". All plants were placed at random positions in the respective climate chamber (B. nigra, LD 12:12; S. lycopersicum, LD 16:8) and the trays were moved to new random positions every 3 days.

For wild queen damaging assays:
To assess the leaf-damaging behavior of different species we collected wild queens in a field season starting from February 2023-July 2023. We collected wild individuals at different field sites in Switzerland of varying elevations (from 405 m a.s.l to 2000 m a.s.l). We used insect nets to collect individuals and kept only the ones without pollen-bags, indicating that they had not yet established a nest. We stopped collecting as soon as we observed flying workers at each collection sites. After identification, individuals were kept in small enclosures (W30 x D30 x H30) in a climate chamber together with flowerless plants of Brassica nigra (20-30 days post germination) or Solanum lycopersicum (30-40 days post germination) (Kälte 3000, light/dark cycle: 16:8, 150 µE; RH60%/70%; 22/18°C). The plant species a queen received was based on availability. Plants were inspected daily for new leaf-damage. Damage was documented by cutting damaged leaves and scanning them using the Epson Perfection V850 Pro Scanner. The following settings were applied in the Epson Scan Software: document type: film; film type: color positive film; image type: 24-bit color; resolution: 1200 dpi; adjustments: none; file type: TIFF. Leaves were scanned in batches every 2-3 days.

For leaf-damaging assays with wild workers:
For our microcolony experiments, we collected wild foraging workers from different species in May 2023. 10-20 worker per species, originating from different colonies, were collected in the center of Zurich, kept together in a plastic box with sufficient ventilation in a climate chamber (Kälte 3000: day/night cycle: 16:8, 24h darkness; RH60%/70%; 22/18°C), and given around 10 days to establish a new hierarchy. The only species that successfully formed a working microcolony was Bombus pratorum and was placed in a cage (W60 x D60 x H60 cm) in a climate chamber (Kälte 3000: light/dark cycle: 16:8, 150 µE; RH60%/70%; 22/18°C) and presented with flowerless S. lycopersicum plants. Damaged plants were removed from the cage and the leaves were scanned as described above.

Timing and spatial scale

For flowering time recording:
Plants were monitored daily for the development of macroscopic signs of flowering (B. nigra, first flowering primordium; S. lycopersicum, first open flower), and flowering time was assessed as "time (days) elapsed since damage treatment".

For queen collection:
Wild bumblebee queens were caught using insect nets in Spring 2023 (end of February 2023 until end of July 2023). We collected queens from different regions of Switzerland and from different elevations. Because of the delayed phenology at higher elevation, which shifted the timing of queen emergence to mid-July (Snow melt dates for 2000 m asl: mid-May), we were able to prolong our field season. To avoid collecting queens that had already initiated a colony, we only kept queens without pollen bags and stopped collecting queens as soon as we observed flying workers at each collection site.

Damaging Assays:
Individual queens were kept in small enclosures (W30 x D30 x H30 cm) in a climate chamber for at least 2 weeks and given access to nectar ad libitum. Pollen was provided once a week, but during exposure to plants no pollen was available to ensure conditions of pollen-deprivation. Plants were checked daily for leaf-damage.

Data exclusions

Some individuals of wild queens were excluded from the damaging assays for two reasons:
- either they were directly used for rearing purposes, meaning they never participated in a damaging experiment (see label "direct in box")
- or the individual died overnight (within 24h after collection).
This can be seen in the excel sheet Bombus_queen_collection_list.xlsx in the data repository.

Reproducibility

The damaging activity of Bombus terrestris queens was observed multiple times in several different experiments, confirming, that it is reproducible and consistent. For the wild species, a second field season is planned to reproduce the behavior in some key species. The flowering-time effect was performed once, and because of the lack of queens not yet reproduced. However, we know it from several experiments with workers, that the effect is consistent and reproducible.

Randomization

Plants were assigned randomly to queens in both the flowering-time study and the damaging assays. For the flowering-time study, each damaged plant was then paired with a mechanical damage control, where we tried to find plants with similar leaf number to the queen-damaged one.

Blinding

No blinding was used for the data analysis.

Did the study involve field work? ☒ Yes ☐ No

Field work, collection and transport

Field conditions	Wild bumblebee queens were caught using insect nets in Spring 2023 (end of February 2023 until end of July 2023). Delayed phenology at higher elevation shifted the timing of queen emergence to mid-July (Snow melt dates for 2000 m asl: mid-May).
Location	We collected queens from different regions of Switzerland and from different elevations: Haldenstein (Calanda 1400 m a.s.l., 2000 m a.s.l.), greater Zurich area (City Center 400 m a.s.l., Uetliberg 870 m a.s.l.), Baden 500 m a.s.l., Landquart 550 m a.s.l., Niederwil 405 m a.s.l..
Access & import/export	For our fieldwork no bumblebees were imported to / or exported from Switzerland.<br>For our commercial bumblebees: we imported them via a local distributor from Belgium (Biobest group NV, Westerlo).
Disturbance	To avoid collecting queens that had already initiated a colony, we only kept queens without pollen bags and stopped collecting queens as soon as we observed flying workers at each collection site.

Reporting for specific materials, systems and methods

We require information from authors about some types of materials, experimental systems and methods used in many studies. Here, indicate whether each material, system or method listed is relevant to your study. If you are not sure if a list item applies to your research, read the appropriate section before selecting a response.

Materials & experimental systems

n/a	Involved in the study	
☒	☐	Antibodies
☒	☐	Eukaryotic cell lines
☒	☐	Palaeontology and archaeology
☐	☒	Animals and other organisms
☒	☐	Clinical data
☒	☐	Dual use research of concern

Methods

n/a	Involved in the study	
☒	☐	ChIP-seq
☒	☐	Flow cytometry
☒	☐	MRI-based neuroimaging

Animals and other research organisms

Policy information about studies involving animals; ARRIVE guidelines recommended for reporting animal research, and Sex and Gender in Research

Laboratory animals	This study did not involve laboratory animals.
Wild animals	Individual spring queens were caught in the field using an insect net in early spring 2023. Caught queens were stored in jars, with holes in the lids, to ensure proper ventilation. The jars were immediately placed in a dark cool box, including pre-cooled ice-packs, to minimize stress. The queens were provided with a small amount of nectar before bringing them back to the laboratory. After the experiments, queens were subjected to either:<br>- Rearing. Queens of selected key species were kept after the experiments to try and rear a colony.<br>- Release. In case, a field trip followed shortly on end of the experiment, queens were brought back to their collection site and released.<br>- Freezing. In case, an individual was not used for further experiments and could not be released within reasonable time, they were killed by freezing. Those specimens are being prepared to be donated to our local insect collection.<br><br>A list of caught species is provided here.<br>Bombus terrestris<br>Bombus mendax<br>Bombus soroeensis<br>Bombus hortorum<br>Bombus sylcarum<br>Bombus pascuorum<br>Bombus mesomelas<br>Bombus humilis<br>bombus vestalis<br>Bombus sylvestris<br>Bombus spp.<br>Bombus rupestris<br>Bombus barbutellus<br>Bombus pratorum<br>Bombus monticola<br>Bombus jonellus<br>bombus terrestris

	Bombus lapidarius<br>Bombus wurflenii<br>Bombus ruderatus<br><br>For the microcolony experiment:<br>Wild Bombus pratorum, Bombus humilis and Bombus pascuorum workers were caught in close vicinity to our laboratory, therefore there was no transportation necessary. In case of B. humilis and B.pascuorum workers were frozen, after they failed to establish a microcolony within 2 weeks. In case of B. pratorum the microcolony was used for a damaging assay and after used for other behavioral experiments, before eventually being killed by freezing.
Reporting on sex	Findings only apply to female bumblebees of two castes: queens, of different developmental stages, and workers.
Field-collected samples	Individual queens were kept in small enclosures (W30 x D30 x H30 cm) in a climate chamber (Kälte 3000: light/dark cycle: 12:12, 150 µE; RH60%/70%; 22/18°C) for at least 2 weeks and given access to nectar (Biogluc sugar solution, Biobest group) ad libitum. Pollen (Bio-Blütenpollen, naturwaren-niederrhein GmbH, Germany) was provided once a week, but during exposure to plants no pollen was available to ensure conditions of pollen-deprivation.
Ethics oversight	In Switzerland, there is no legislation regulating research with bumblebees; however, our experimental design and procedures were guided by the 3R principles29. Bees received daily care by trained staff and were provisioned with adequate food. Behavioral tests were non-invasive, and we tried to minimize stress wherever possible.

Note that full information on the approval of the study protocol must also be provided in the manuscript.
